# Down-Regulation of *PU.1* Gene in Pediatric Acute Lymphoblastic Leukemia Patients from South of Iran

**Published:** 2019-01-01

**Authors:** Bita Nakhost, Mahboobeh Nasiri, Mehran Karimi, Somayeh Montazeri

**Affiliations:** 1 Department of Biology, Islamic Azad University, Arsanjan Branch, Arsanjan, Iran; 2 Hematology Research Center, Shiraz University of Medical Sciences, Shiraz, Iran; 3 Thalassemia and Hemophilia Genetic, PND Research Center, Dastgheib Hospital, Shiraz University of Medical Sciences, Shiraz, Iran

**Keywords:** Pediatric ALL, *PU.1*, Gene expression

## Abstract

**Background: **Acute lymphoblastic leukemia (ALL) is resulted from the infiltration of high amount of non-differentiated cells in bone marrow. Differentiation of the hematopoietic stem cells into specific cell lineage occurs through a highly regulated pathway which is mainly monitored during transcription step. Expression level and pattern of transcription factors e.g. PU.1 determine fate and developmental phases in this pathway. This study was performed to evaluate the expression level of the *PU.1* gene in a group of children suffering from ALL.

**Materials and Methods: **The mRNA expression level of the *PU.1* gene was compared between 30 children diagnosed as new cases of ALL and 30 sex- and gender-matched healthy children in the present case-control study. The quantitative real time PCR (qRT-PCR) was used to determine the level of *PU.1* gene expression. The data were analyzed using Graph Pad Prism statistical software.

**Results: **The mRNA level of the *PU.1* gene was significantly lower in the blood samples of the ALL patients compared to the controls (*p*= 0.002).

**Conclusion: **The results of the study indicated that the *PU.1* gene seemed to have key roles in the differentiation pathway of blood cells.

## Introduction

 All blood cell types originate from multi-potent hematopoietic stem cells (HSCs) through a highly orchestrated process, called hematopoiesis^   1 ^. Any disturbance in this highly conserved critical process deregulated the cell decision for proliferation or differentiation which may result in human cancers such as leukemia^   2 ^. Among all types of leukemia, acute lymphoblastic leukemia (ALL) is the most common malignancy in young children. It is characterized by a block of B- and T-cell differentiation and the accumulation of immature B and T lymphoblast, which are called B-lymphoblastic ALL and T-cell ALL, respectively^   3 ^. 

Transcriptional regulation seems crucial for maintaining the sequential stages of the hematopoiesis since involving in the programming of cell fate and their lineage commitment by regulating lineage-specific gene expression ^4^^,^^5^ . The transcription factor, purine-rich box1 (PU.1 or Spi-1), belongs to the E26 transformation-specific (ETS) family and shows to have a critical role in the health of hematopoiesis^        6 ^. *PU.1* gene is differentially expressed in various hematopoietic cells and regulates hematopoiesis in dose-dependent manner^        6 ^. The expression level of *PU.1* represents an ascending gradient toward the development of lymphoblast to mature B-cells, but shows attenuation toward the more committed T cells ^7^^-^^9^ .

Regarding previous reports on the influence of altered expression of *PU.1* on erythroleukemia ^   10 ^ and myeloid leukemia^   11 ^, we aimed to clarify whether the *PU.1* gene expression modification affects B-and T-cell lymphopoiesis and involves in acute lymphoblastic leukemia (ALL) malignancy.

## MATERIALS AND METHODS

**Subjects**

This case-control study included 30 new cases of pediatric ALL (mean ± SD: 8.03 ± 3.99 years; 2-17 years) hospitalized in Amir Oncology Hospital, Shiraz, Iran. Twenty-one out of 30 patients were diagnosed as having B- lymphoblastic ALL and the 9 remaining patients were diagnosed with T-cell ALL. Immunology and cytogenetic tests and microscopic observation of the cell morphology were used to confirm the diagnosis. Patients with the age more than 20 years, personal history of any bleeding disorders or other malignancies, history of radiotherapy or chemotherapy at the time of sampling or earlier time, and relapsed ALL were excluded from the study. Thirty healthy subjects (7.86 ± 4.15 years) matched by age and gender were also investigated as a control group. According to all parents in an oral interview, none of the children had prior history of malignancies or bleeding disorders. Written informed consent was signed by all parents (cases and controls) and the procedures of the project were definitely explained to those parents who were interested in detailed information. The study was approved by the Institutional Review Board of our department.

**RNA extraction, cDNA synthesis, and Real-Time PCR analysis**

Total RNA was extracted from 2-4 ml of fresh blood using RNX-Plus solution (CinnaGen, Iran) following the manufacturer’s protocol. cDNA was synthesized using RevertAid first-strand cDNA synthesis kit (Thermo Scientific Fermentas, USA) according to the manufacturer’s instructions. Primer pairs for *PU.1* and TATA-binding protein (*TBP*) as an internal control gene were designed by Allele ID v7.8 software. Primer sequences were as follows: forward, 5′-TTACCCCTATCTCAGCAG-3′ and reverse, 5′-GAAGTTGTTCTCGGCGAAG-3′ for *PU.1* gene; and forward, 5′-CCCGAAACGCCGAATATAAT-3′ and reverse, 5′-CTGGACT GTTCTTCACTCT TG-3′ for *TBP* gene. The quantitative real-time PCR was performed in the Rotor-Gene Q 2plex HRM Platform real-time PCR system (Corbett Life Science) machine in the volume of 15 µl containing 7.5µl of 2x SYBR-Green master mix (Yekta Tajhiz Azma, Iran), 5µl of cDNA, 0.4µl and 1.5µl of each primer pair for *TBP* and *PU.1* genes. All reactions were done in duplicate. The PCR condition was as follows: initial denaturation at 95°C for 15 min followed by 40 cycles of denaturation at 95°C for 15 s; annealing at 58°C for 60 s and extension at 72°C for 60 s. The Threshold Cycle (CT) values were determined and the relative expression levels of the target gene were normalized to the expression levels of the endogenous control gene, TBP. The data were analyzed using the comparative threshold cycle (2^-∆∆CT^) method.

**Statistical analysis**

Expression level difference was evaluated using GraphPad Prism statistical software (La Jolla, USA). Unpaired t-test was used to show the difference in gene expression between cases and controls. Nominal variables were presented as mean± standard deviation (SD). The numbers and percentages were directly calculated and presented for each of the categorical variables, and then compared between cases and controls using Pearson Chi-square test. The *p*-value equivalent or less than 0.05 was considered statistically signiﬁcant for all types of analyses. 

## Results

 Baseline characteristics of the study population are presented in Table 1. The disease was more frequent in males than in females (63.3% vs. 36.7%). 

**Table 1:** General characteristics of the study population

**Characteristics**	**Controls **	**ALL cases**
Number	30	30
Age (mean±SD; years)	7.86± 4.15	8.03± 3.99
Gender (M/F)	19/11	19/11
ALL type		
B-lymphoblastic	-	21
T-ALL	-	9

**PU.1 gene expression analysis**

**Figure 1:**
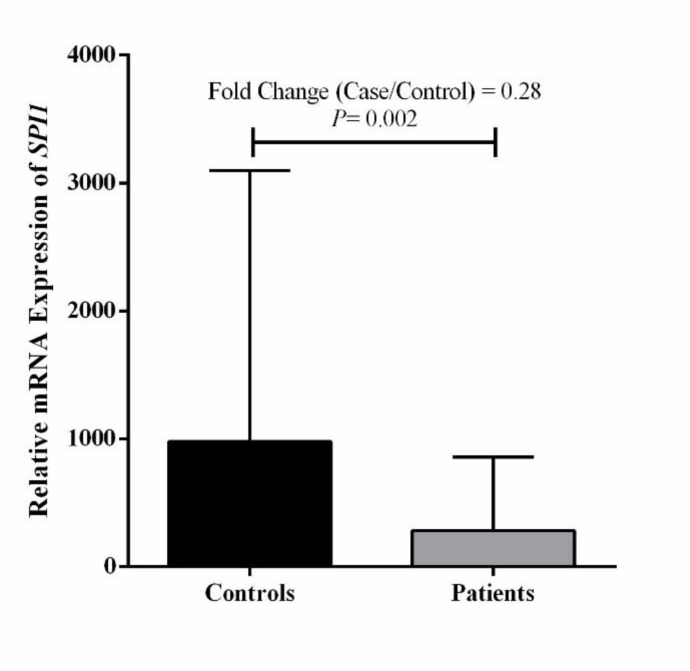
Expression status of *PU.1* gene in ALL patients and controls

Comparison of the expression level of the *PU.1* gene between children with ALL and healthy children resulted in the significant difference (*p*=0.002). The reduced expression level of *PU.1* gene was observed in ALL group compared with the control group (Figure 1). 

## Discussion

 To the best of our knowledge, this is the first report of investigating the expression level of *PU.1* gene in pediatric ALL patients in Iran. The results are more considerable since all the ALL patients were described as new cases. Down-regulation of *PU.1* gene in our patients creates new insight into the importance of transcription regulation in the pathogenesis of ALL and introduces this gene as a probable molecular target in designing new treatment strategies.

Normal hematopoiesis is securely controlled by a small number of lineage-specific transcription factors, so that the disturbed expression or function of this group may be involved in the development of leukemia ^   12 ^. PU.1 acts as an upstream regulator for the transcription factors c-Jun and b-Jun ^   13 ^. Multiple knockdown models have demonstrated the importance of *PU.1* gene in the susceptibility to ALL ^   11 ^, while more frequency of myeloid leukemia has been seen in *PU.1*-deficient adult mice^   14 ^. Evaluating the pre-leukemic hematopoietic stem cells (pHSCs) transcript in response to *PU.1* knockdown resulted in the obvious changes in the c- and b-Jun transcription factors^   13 ^. Polgárová et al. (2016) recently published a list, containing 90 regulatory and differentiation-related genes e.g. *PU.1* for their involvement in the human hematopoiesis^   15 ^. Despite previous studies on the reduced expression of the *PU.1* gene in the CML patients, the direct investigation of *PU.1* mRNA level in the blood samples of the ALL patients was not performed  ^16^^, ^^17^ . But the reduced PU.1 expression was evaluated in bone marrow cells of ALL patients and the related mice models, with a presumption of AML1-ETO involvement ^   18 ^ or Notch signaling ^   19 ^. Heterozygous deletion of the *PU.1* locus was supposed to be associated with human AML. In two distinct studies, 126 and 120 AML patients were analyzed using direct sequencing ^20^^,^^21^ **.** Quantitative real-time PCR; however, revealed a decrease in gene expression^   21 ^. Aside from mutations, a SNP in a distal enhancer of *PU.1* gene was also reported a reduction in the *PU.1* gene expression^   22 ^. Although it was out of our research scope to look for rare *PU.1* mutations in human samples, the lower expression of the gene was observed in our study. A similar finding was also observed in acute promyelocytic leukemia (APL) and considered to be in correlation with APL initiation ^   23 ^ and progression ^   24 ^. The most recent attempt for elucidating the mechanism of PU.1 protein provided this idea that PU.1 supports TRAIL-induced cell death by affecting an NF-κB-related pathway in AML^   25 ^, or a trichostatin A-induced apoptosis in collaboration with Bim during lymphoma^   26 ^. We suggest other researchers share their clinical data along with their genetic analysis for *PU.1* and other newly discovered genes. Here, we reported that the transcriptional level of *PU.1 *gene is significantly lower in Iranian pediatric patients with ALL, and more studies with larger sample sizes are required to confirm this finding by providing novel PU.1 associated mechanisms. 
